# Human breast cancer cell lines contain stem-like cells that self-renew, give rise to phenotypically diverse progeny and survive chemotherapy

**DOI:** 10.1186/bcr1982

**Published:** 2008-03-26

**Authors:** Christine M Fillmore, Charlotte Kuperwasser

**Affiliations:** 1 Department of Genetics, Sackler School, Tufts University School of Medicine, Harrison Avenue, Boston, Massachusetts 02111, USA; 2 Department of Anatomy & Cellular Biology, Sackler School, Tufts University School of Medicine, Harrison Avenue, Boston, Massachusetts 02111, USA; 3 Department of Radiation Oncology, Tufts-New England Medical Center, Molecular Oncology Research Institute, Boston, Massachusetts 02111, USA

## Abstract

**Introduction:**

The phenotypic and functional differences between cells that initiate human breast tumors (cancer stem cells) and those that comprise the tumor bulk are difficult to study using only primary tumor tissue. We embarked on this study hypothesizing that breast cancer cell lines would contain analogous hierarchical differentiation programs to those found in primary breast tumors.

**Methods:**

Eight human breast cell lines (human mammary epithelial cells, and MCF10A, MCF7, SUM149, SUM159, SUM1315 and MDA.MB.231 cells) were analyzed using flow cytometry for CD44, CD24, and epithelial-specific antigen (ESA) expression. Limiting dilution orthotopic injections were used to evaluate tumor initiation, while serial colony-forming unit, reconstitution and tumorsphere assays were performed to assess self-renewal and differentiation. Pulse-chase bromodeoxyuridine (5-bromo-2-deoxyuridine [BrdU]) labeling was used to examine cell cycle and label-retention of cancer stem cells. Cells were treated with paclitaxol and 5-fluorouracil to test selective resistance to chemotherapy, and gene expression profile after chemotherapy were examined.

**Results:**

The percentage of CD44^+^/CD24^- ^cells within cell lines does not correlate with tumorigenicity, but as few as 100 cells can form tumors when sorted for CD44^+^/CD24^-/low^/ESA^+^. Furthermore, CD44^+^/CD24^-^/ESA^+ ^cells can self-renew, reconstitute the parental cell line, retain BrdU label, and preferentially survive chemotherapy.

**Conclusion:**

These data validate the use of cancer cell lines as models for the development and testing of novel therapeutics aimed at eradicating cancer stem cells.

## Introduction

The process of wound healing to replace damaged tissue involves epithelial tissue regeneration; a small population of replenishing stem cells gives rise to differentiated progeny that replace the damaged tissue. This characteristic of accelerated epithelial tissue regeneration is shared by the epithelial component in a growing tumor, albeit on a genetically unstable background. In fact, epithelial tumors have been described as 'wounds that do not heal' because of the molecular and cellular similarities between the mesenchyme associated with wounds and that of carcinomas [1]. Many solid tumor types, including breast cancer, exhibit a functional hierarchy of cancer cells of which only a small subpopulation of replenishing stem-like cells can give rise to the differentiated cells that comprise the bulk tumor [2-6]. In human breast cancers, these tumorigenic breast cancer stem cells are enriched in cells with a CD44^+^/CD24^-/low^/ESA^+ ^phenotype [2].

Other than the ability to seed a tumor in a nonobese diabetic (NOD)/severe combined immunodeficient (SCID) mouse, it is unclear what phenotypic and functional differences distinguish the cells that fuel carcinoma growth from cells that comprise the tumor bulk. Furthermore, it is unclear what mechanisms control the maintenance and survival of these tumorigenic cells. These issues have been difficult to study, in part because of the presupposed lack of appropriate model systems. Use of primary breast cancer cells is considered to be the best means to study tumor repopulation because it is presumed that, upon long-term cultivation *in vitro*, cancer cells lose the dynamic characteristics of a regenerating tissue [7]. However, experiments with primary tumor cells are costly and difficult to control because of small sample size and the heterogeneous nature of the cellular, genetic, and epigenetic composition among patient tissue samples.

In order to overcome problems associated with procuring and using primary human tissues, continuous breast cancer cell lines have been developed from various sources, including pleural effusions (MDA.MB.231 and MCF7), primary breast cancers (SUM149 and SUM159), primary tumor recurrences (SUM225), and even xenografted metastatic nodules (SUM1315) [8,9]. These cell lines represent polyclonal populations of cells that have adapted to tissue culture conditions but retain many of their phenotypic and genotypic properties over countless passages [9,10]. Genomic approaches have revealed that, like primary tumors, the gene expression signatures of breast cancer cell lines can distinguish luminal from basal subtypes of breast cancer [11-13]. Moreover, cell line derived gene signatures can correctly classify human tumor samples [8,14]; this suggests that, despite their acquired ability to grow *in vitro*, cell lines continue to share many of the molecular and genetic features of the primary breast cancers from which they were derived.

Breast cancer cell lines will form tumors in immunodeficient mice that recapitulate the histology, progression, and metastatic spread of the disease [15-17]. Therefore, we postulated that cancer cell lines may also retain the cellular hierarchy characteristic of primary breast tumors. In this study, we report that breast cancer cell lines contain a small population of cells that mimic cancer stem cell behaviors. Similar to primary breast cancers, cell line derived tumor-initiating cells are enriched in cells with the CD44^+^/CD24^-/low^/epithelial-specific antigen (ESA)^+ ^phenotype. This work validates the use of cell lines to elucidate the unique mechanisms that govern maintenance and survival of tumorigenic breast cancer stem cells.

## Materials and methods

### Cell lines and tissue culture

SUM cell lines were obtained from Dr Stephen Ethier (Kramanos Institute, MI, USA) and are commercially available (Asterand, Detroit, MI); the MDA.MB.231, MCF7, and MCF10A cell lines were purchased from American Type Culture Collection (Manassas, VA, USA). Human mammary epithelial cells immortalized with the catalytic subunit of telomerase [18] were cultured in Dulbecco's modified Eagle's medium-F12 (1:1) with 5% calf serum (Hyclone, Logan, UT), insulin (5 μg/ml), hydrocortisone (1 μg/ml), and epidermal growth factor (10 ng/ml; Sigma, St. Louis, MO). MCF10A and MCF7 cells were cultured in Dulbecco's modified Eagle's medium with 10% calf serum. MDA.MB.231 cells were cultured in RPMI 1640 with 10% calf serum. SUM225CWR, SUM149PT, and SUM159PT cells were cultured in Ham's F12 with 5% calf serum, insulin (5 μg/ml), and hydrocortisone (1 μg/ml), whereas SUM1315MO2 were in Ham's F12 with 5% calf serum, insulin (5 μg/ml), and epidermal growth factor (10 ng/ml). All cell lines were grown at 37°C and 5% carbon dioxide.

### Flow cytometry

Nonconfluent cultures were trypsinized into single cell suspension, counted, washed with phosphate-buffered saline (PBS), and stained with antibodies specific for human cell surface markers: ESA-FITC (Biomeda), CD24-PE, and CD44-APC (BD Pharmingen, San Jose, CA). A total of 250,000 cells were incubated with antibodies for 15 minutes at room temperature. Unbound antibody was washed off and cells were analyzed no longer than 1 hr post staining on a BD Facscalibur.

### Animals and surgery

All animal procedures were performed in accordance with an approved protocol by the Tufts University Institutional Animal Care and Use Committee. A colony of NOD/SCID mice was maintained under sterile conditions and received food and water *ad libum*. Nulliparous female mice aged 8 to 14 weeks were utilized in all experiments. Human breast cancer cells were resuspended in 1:1 (vol/vol) media and Matrigel (BD Biosciences, San Jose, CA), and injected into the fourth inguinal mammary gland. Tumor formation was assessed by palpitation at least once a week.

### Tumorsphere assays

A total of 5,600 single cells/cm^2 ^were plated on super-low adherence plates (Corning, Lowell, MA). Every 3 days, after cells pelleting and resuspending in fresh media, spheres visible by eye (no magnification) were counted. Pictures were taken to assess the ratio of spheres to aggregates of cells. To correct for differing growth rates of the cell lines, the average tumorsphere number on day 12 was divided by a proliferation constant for the cell line. To quantify proliferation, cells were seeded on day 0 at 50,000 cells/well in a six-well dish in duplicate. Cells were fixed and stained with 0.1% crystal violet and quantified by extraction in 10% acetic acid and spectrophotometer reading at optical density 600 nm. Spheres were fractionated by size using 100 μm cell strainers (BD Pharmingen) and trypsinized into single cell suspension for flow cytometric analysis, as described above.

### BrdU label retention

Nonconfluent cultures were pulsed with 10 μmol/l bromodeoxyuridine (5-bromo-2-deoxyuridine [BrdU]) for 10 days, being split and re-fed so that a total of 50 μmol/l BrdU was given to no more than 5 × 10^6 ^cells. Chased cells were analyzed at day 0, 4, 6, and 8 days for BrdU label retention. For these pulse-chase studies, at day 0 totals of 10,000, 5,000, or 1,000 BrdU-labeled cells were added per well of a four-well chamber slide and fixed at 4, 6, or 8 days after chase, respectively. Cells chased with chemotherapy were analyzed at day 6. For immunofluorescence, cells were prepared similarly to those described in Shinin and coworkers (2006) using 4% paraformaldehyde and 0.1% saponin in PBS as a fixative; 0.25% Triton-X and 0.1% bovine serum albumin in PBS as a permeabilization buffer; and 0.02 U/μl of DNase (NE Biolabs, Ipswich, MA). Antibodies used were mouse monoclonal anti-BrdU (BD Pharmingen), rabbit polyclonal anti-Ki67 (Abcam, Cambridge, MA), and anti-mouse Alexa 488 (Molecular probes). Slides were mounted in Vecta-shield mounting media with DAPI (4',6 diamidino-2-phenylindole; Vector Laboratories, Burlingame, CA, USA). For flow cytometry, mouse monoclonal anti-EpCAM (Abcam), anti-mouse PerCP, and the BrdU-FITC set of antibodies was used (BD Pharmingen).

### Chemotherapy treatment

Confluent plates were split 1:4 and treated the following day with a 1:100 dilution of 0.1 mol/l 5-fluorouracil (5-FU) in dimethyl sulfoxide (DMSO) for a final concentration of 1 mmol/l 5-FU, or 1 μm paclitaxel in DMSO for a final concentration of 10 nmol/l Taxol (Sigma). Placebo control plates received 1:100 DMSO. Cells were fed and re-treated at 3 days and analyzed at 6 days.

## Results

### Percentage of CD44^+^/CD24^- ^cells associates with a basal phenotype, not tumorigenicity

Breast cancers are broadly categorized into two classes: those that express luminal keratins (luminal-type) and those that express stratified epithelial keratins (basal-type) [11,12,19,20]. Recent work has characterized the cellular heterogeneity of human breast tissues based on cell surface expression of CD44 and CD24, using epithelial cells from primary reduction mammoplasty tissues, pleural effusions, and primary tumors [21]. CD44^+^/CD24^- ^cells exhibit features of basal cells and express genes that are involved in motility, whereas CD24^+^/CD44^- ^cells exhibit features of more differentiated luminal epithelial cells and express genes involved in hormone responses.

**Figure 1:**
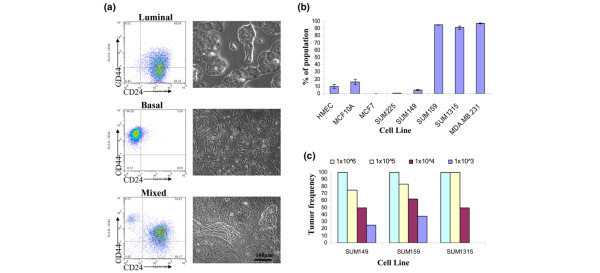
CD44^+^/CD24^- ^cells associate with a basal phenotype not with tumorigenic potential of human breast cancer cell lines. **(a) **Luminal, basal or mixed cell morphology can be predicted based on CD44 and CD24 expression. Provided are representative flow cytometry dot plots of CD44 and CD24 expression adjacent to phase-contrast brightfield images of each cell line subtype. Cell lines are composed of luminal-like, CD24^+ ^expressing cells (MCF7 and SUM225); basal-like, CD24^- ^expressing cells (SUM159, SUM1315, and MDA.MB.231); or mixed luminal and basal-like cells and consist of both CD24^+ ^and CD24^- ^populations (human mammary epithelial cells [HMEC] and SUM149). **(b) **The average percentage of CD44^+^/CD24^- ^cells in each cell line was determined by flow cytometry. Data are presented as the mean ± standard error of the mean for four separate experiments. **(c) **The frequency of tumor growth was measured for SUM149, SUM159, and SUM1315 cell lines injected orthotopically in nonobese diabetic/severe combined immunodeficient mice when a tumor was palpable (>3 mm) within 100 to 150 days.

To examine whether breast cancer cell lines adhere to a similar molecular definition, we examined eight different human breast cell lines with differing tumorigenic, invasive, and metastatic potentials (Additional file 1). Using flow cytometry, cell lines could be could be grouped into three classes based on CD44 and CD24 expression profiles (Figure 1a and Additional file 2). Class 1 (luminal) includes the MCF7 and SUM225 lines that were mainly CD24^+ ^and exhibit a more differentiated morphology and luminal cytokeratin expression, which is consistent with their luminal-type classification (Figure 1a; data not shown) [8,21]. Class 2 (basal/mesenchymal) consists of the MDA.MB.231, SUM159, and SUM1315 lines that are more than 90% CD44^+^/CD24^- ^cells and exhibit a spindle-like appearance, consistent with their basal-type classification. Finally, Class 3 (mixed) consists of the SUM149 and human mammary epithelial cell lines, both of which exhibit two distinct CD44/CD24 populations and contain distinct populations of cells with either basal-like or luminal-like features. These data suggest that cultured breast cell lines maintain a similar cellular definition as cells in primary human tissues.

Cells from primary human breast tumors with a CD44^+^/CD24^-/low ^phenotype enrich for tumor formation *in vivo *[2]. Because basal cell lines comprise more than 90% CD44^+^/CD24^- ^cells, we reasoned these lines should be more tumorigenic than the mixed cell line, SUM149, which contains an average of 18-fold fewer CD44^+^/CD24^- ^cells (Figure 1b). To compare quantitatively the tumorigenicity of the cell lines, we performed limiting dilution injection experiments. SUM149, SUM1315, and SUM159 cell lines were injected into NOD/SCID mice in a 10-fold dilution series from 1 × 10^6 ^to 1 × 10^3 ^cells per mammary gland and the mice were monitored for tumor growth (Figure 1c). Despite containing more than 90% CD44^+^/CD24^- ^cells, the SUM1315 and SUM159 lines form tumors only 50% or 60% of the time with 10,000 cells injected, respectively. Surprisingly, SUM149 line, which contains only about 5% CD44^+^/CD24^- ^cells, exhibits a tumorigenic potential similar to that of the SUM159 and SUM1315 lines. Collectively, these data suggest that, analogous to primary breast cancers [21], a basal cellular phenotype rather than tumorigenicity consistently correlates with the percentage of CD44^+^/CD24^- ^cells in cell lines.

### Breast cancer cell lines contain CD44^+^/CD24^-^/ESA^+ ^breast cancer stem-like cells

**Figure 2:**
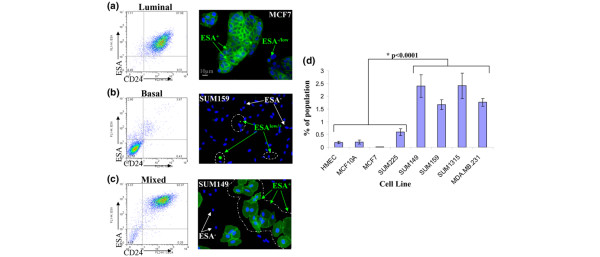
ESA can distinguish luminal, basal, and stem-like cells. Presented are representative flow cytometry dot plots of epithelial-specific antigen (ESA) and CD24 expression adjacent to ESA immunofluorescence of each cell line subtype. Cell lines are as follows: **(a) **luminal-like, expressing high levels of ESA and CD24 (MCF7, SUM225, and T47D); **(b) **basal-like, expressing low levels of ESA and CD24 (SUM159, SUM1315, and MDA.MB.231); or **(c) **mixed luminal/basal cultures that contain both ESA^+^/CD24^+ ^and ESA^-^/CD24^- ^populations (SUM149). **(d) **The average percentage of CD44^+^/CD24^-^/ESA^+ ^cells in each cell line, as determined by flow cytometry. Data are presented as the mean ± stamdard error of the mean for five separate experiments. HMEC, human mammary epithelial cells.

Because the percentage of CD44^+^/CD24^- ^cells in breast cancer cell lines does not predict tumorigenic potential, we next considered whether adding ESA to the repertoire of cell surface markers could enrich for tumor initiation capacity, as was previously demonstrated [2]. ESA and CD24 are coexpressed in malignant breast cell lines, such that ESA expression can substitute for CD24 expression in classifying the lines into luminal, basal, and mixed phenotypes (Figure 2a–c). The luminal lines, MCF7 and SUM225, exhibit two populations of ESA-expressing cells; the majority express high levels of ESA, as indicated by immunofluorescence and flow cytometry (ESA^+^), whereas a smaller subset of cells express a lower level (ESA^low^; Figure 2a). Conversely, the basal lines SUM159, SUM1315, and MDA.MB.231 contain a large population of ESA-negative (ESA^-^) cells and a smaller subset of cells expressing low levels ESA (ESA^low^; Figure 2b). Finally, mixed cell lines such as SUM149, which contains both basal and luminal cell types, exhibit three ESA populations: those that are negative for ESA (ESA^-^) and two populations of ESA-positive cells, namely ESA^+ ^and ESA^low ^cells (Figure 2c).

Using flow cytometry, CD44^+^/CD24^-^/ESA^+ ^cells were detected in all of the lines examined (Figure 2d). The percentage of CD44^+^/CD24^-^/ESA^+ ^cells ranges from 0.01% to 0.5% in luminal lines, such as MCF7 and SUM225, to about 2.5% in the basal and mixed cell lines SUM149, SUM159, SUM1315, and MDA.MB.231 (Figure 2d). Most importantly, there is a clear correlation (*P *< 0.0001) between the percentage of CD44^+^/CD24^-^/ESA^+ ^cells in a cell line and the tumorigenic and aggressive phenotype of the line *in vivo *(Additional file 1 and Figure 2d).

**Figure 3:**
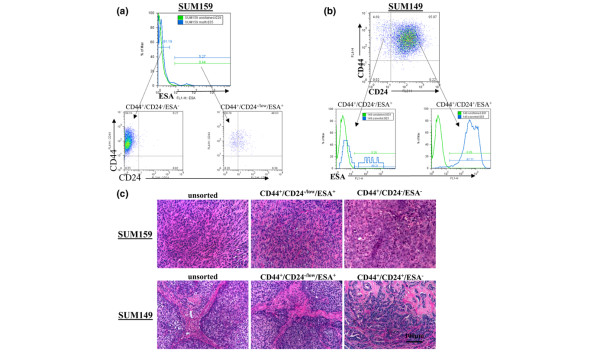
CD44^+^/CD24^-/low^/ESA^+ ^cells in human breast cancer cell lines enrich for tumorigenicity. Shown are representative flow cytometry analysis and gates for epithelial-specific antigen (ESA), CD24 and CD44 sorting. **(a) **SUM159 cells were sorted on ESA. Dot plots show resulting CD44^+^/CD24^-/low^/ESA^+ ^or CD44^+^/CD24^-^/ESA^- ^fractions. **(b) **SUM149 cells were sorted on CD44 and CD24 and subsequently sorted for ESA^+^, shown in histograms. **(c) **Histological analysis of SUM159 and SUM149 orthotopic tumors. The upper panels present representative hematoxylin and eosin stained sections at 20× magnification of tumors that formed from 5 × 10^5 ^unsorted, 1,000 CD44^+^/CD24^-/low^/ESA^+^, or 1 × 10^6 ^CD44^+^/CD24^-^/ESA^- ^SUM159 cells. Note that the tumor histology from CD44^+^/CD24^-^/ESA^- ^sorted cells is different than that from unsorted or CD44^+^/CD24^-/low^/ESA^+ ^cells. The lower panels present hematoxylin and eosin stained sections at 20× magnification of tumors that formed from 1 × 10^5 ^unsorted, CD44^+^/CD24^-/low^/ESA^+^, or CD44^+^/CD24^+^/ESA^+ ^sorted SUM149 cells. Note the tumor histology from CD44^+^/CD24^+^/ESA^+ ^sorted cells is markedly different from that of the unsorted or the CD44^+^/CD24^-/low^/ESA^+ ^sorted cells.

We hypothesized that CD44^+^/CD24^-/low^/ESA^+ ^cells are enriched for tumorigenic potential as compared with the bulk of the cell line. Therefore, SUM159 and SUM1315 lines were sorted for CD44^+^/CD24^-/low^/ESA^+ ^and CD44^+^/CD24^-^/ESA^- ^cells (Figure 3a), whereas the SUM149 cell line was sorted for CD44^+^/CD24^-/low^/ESA^+ ^and CD44^+^/CD24^+^/ESA^+ ^cells (Figure 3b); all sorted fractions were injected into NOD/SCID mice as a 10-fold dilution series from 1 × 10^6 ^to 1 × 10^2 ^cells per mammary gland.

With as few as 100 cells, CD44^+^/CD24^-/low^/ESA^+ ^subpopulations from SUM159 and SUM149 cell lines could form tumors in NOD/SCID mice, whereas unsorted, CD44^+^/CD24^-^/ESA^-^, or CD44^+^/CD24^+^/ESA^+ ^fractions were unable to do so at the same dilution (Table 1). Similar results were seen with 1,000 cells injected for the SUM1315 line. Histological examination of tissue sections reveals that unsorted SUM159 cells form highly invasive spindle-cell carcinomas, and this spindle-cell morphology is recapitulated in SUM159 tumors derived from 1 × 10^3 ^CD44^+^/CD24^-/low^/ESA^+ ^cells (Figure 3c). Tumors could from with as many as 1 × 10^6 ^SUM159 CD44^+^/CD24^-^/ESA^- ^cells, but these tumors lacked the morphology of the parentally derived tumors in all sections investigated (Figure 3c). The tumor histology of unsorted SUM149 cells resembles high-grade carcinomas that incorporate mouse stroma (Figure 3c). This morphology is only recapitulated in tumors derived from the CD44^+^/CD24^-/low^/ESA^+ ^sorted cells (Figure 3c). Examination of the injection sites where CD44^+^/CD24^+^/ESA^+ ^and CD44^+^/CD24^-/^/ESA^- ^cells were unable to form tumors revealed viable cells and Matrigel at the site of injection (data not shown), confirming that lack of tumor growth from these cells was not due to clearing or cell death.

**Table 1:** Limiting dilution tumor formation of CD44^+^/CD24^-/low^/ESA^+ ^sorted cells *in vivo*

Cell type	Days	Number injected and tumors formed				
		
		1 × 10^6^	1 × 10^5^	1 × 10^4^	1 × 10^3^	1 × 10^2^
SUM159 unsorted	13–100	5/5	5/6	5/8	3/8	0/2
SUM159 CD44^+^/CD24^-^/ESA^-^	20–100	8/10	2/2	1/2	0/8	0/10
SUM159 CD44^+^/CD24^-/low^/ESA^+^	20–100	-	-	2/2	6/6	6/10
						
SUM1315 unsorted	20–150	2/2	2/2	2/4	0/4	-
SUM1315 CD44^+^/CD24^-^/ESA^-^	25–150	3/4	2/2	0/4	0/10	-
SUM1315 CD44^+^/CD24^-/low^/ESA^+^	20–150	-	-	3/4	7/8	-
						
SUM149 unsorted	19–150	4/4	3/4	2/4	1/4	0/2
SUM149 CD44^+^/CD24^+^/ESA^+^	20–150	-	2/2	0/4	0/4	0/6
SUM149 CD44^+^/CD24^-/low^/ESA^+^	48–150	-	2/2	2/4	6/6	5/6

ESA, epithelial-specific antigen.

Although tumors will form if more than 100,000 CD44^+^/CD24^-/low^/ESA^- ^and CD44^+^/CD24^+^/ESA^+ ^cells are injected, only tumors from CD44^+^/CD24^-/low^/ESA^+ ^cells, even with as few as 100 cells, phenocopy parentally derived tumors. Therefore, cells from the parental cell line that preferentially seed tumors segregate with the CD44^+^/CD24^-/low^/ESA^+ ^subpopulation. Collectively, these data show that breast cancer cell lines contain cells with the capacity to form large tumors from few seeding cells. Moreover, these resulting tumors recapitulate the cellular heterogeneity of tumors derived from the unsorted parental cell line. Hence, similar to the case in primary tumors, these tumorigenic breast cancer stem-like cells are enriched 100-fold in the CD44^+^/CD24^-/low^/ESA^+ ^fraction across multiple cell lines. (Additional file 3).

### CD44^+^/CD24^-^/ESA^+ ^cells can self-renew and reconstitute the parental cell line

The ability to both self-renew and differentiate into heterogeneous cell types is the definition of a stem cell that is thought to be functionally mimicked by cancer stem cells [7,22-25]. The only way to distinguish between highly proliferative progenitors and stem cells is the ability of the latter to grow indefinitely. However, because established cancer cell lines, by definition, have the ability to grow indefinitely, serial *in vivo *passaging may not necessarily accurately assess self-renewal of stem-like cells. Because serial colony-forming unit (CFU) assays have been used to assay stem cell self-renewal in other systems [26], we examined whether sorted CD44^+^/CD24^-^/ESA^+ ^cells contain more serial CFUs than other subpopulations. To this end, SUM159 and SUM149 cells were sorted into CD44^+^/CD24^-^/ESA^+^, CD44^+^/CD24^-^/ESA^-^, and CD44^+^/CD24^+^/ESA^+ ^populations and seeded at low density. Six days later, colonies larger than 30 cells were quantified in order to determine which fraction of cells exhibits the greatest CFU activity.

**Figure 4:**
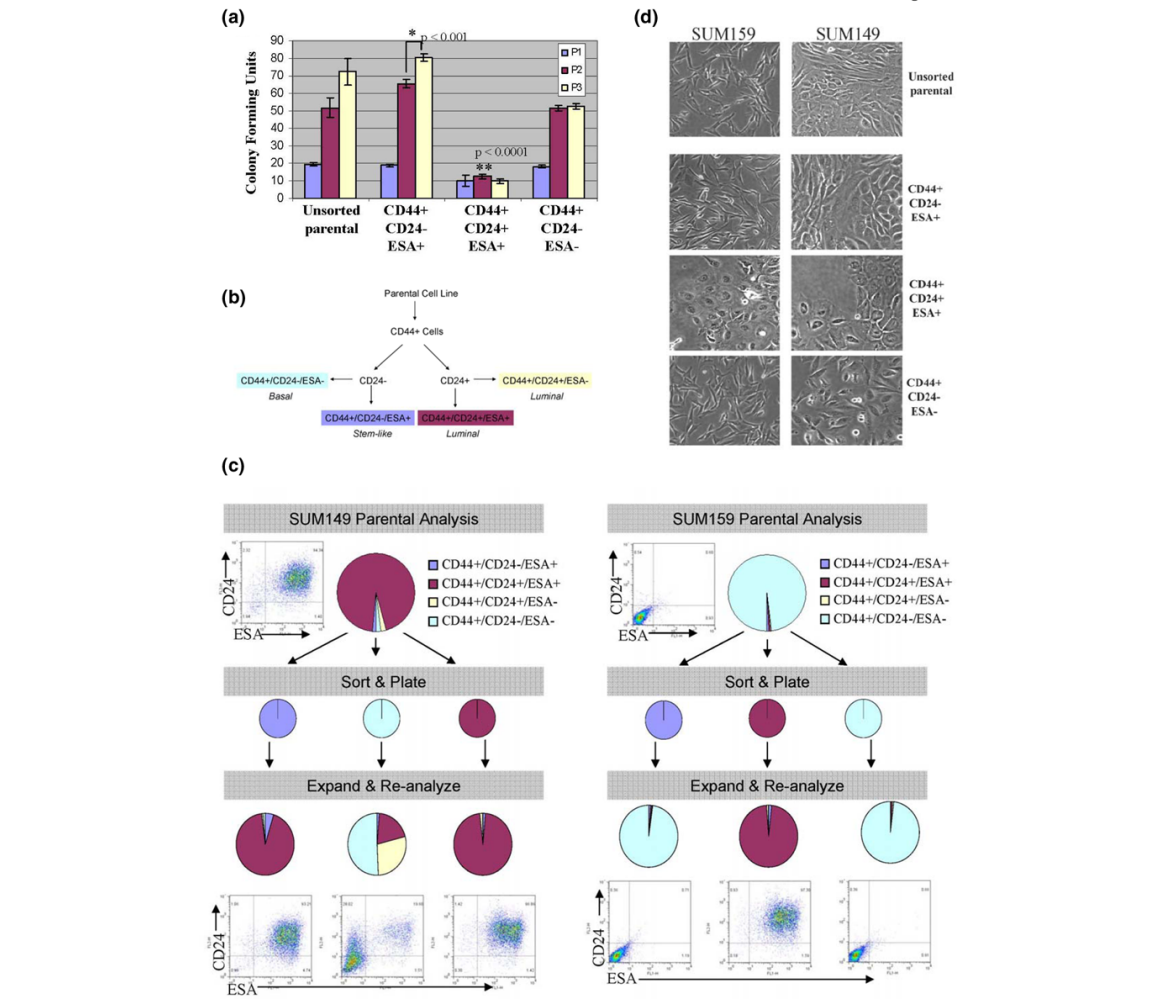
CD44^+^/CD24^-^/ESA^+ ^cells are enriched for self-renewal activity *in vitro*. **(a) **Serial colony-forming units (CFUs) from 300 SUM159 sorted cells. Data are present as mean ± standard error of the mean for three separate experiments. Exhaustion of CFU is defined as the P3 colonies = P2 colonies. **(b) **Flow chart describing sorting scheme for reconstitution. **(c) **SUM149 or SUM159 sorted cells were expanded and the cellular outgrowths were reanalyzed by flow cytometry to assay reconstitution of the parental line. The parental line was run through the flow cytometer and expanded in parallel to control of the effects of fluorescence-activated cell sorting (top).**(d) **Representative phase-contrast brightfield images of unsorted SUM159 or SUM149, and colonies that grew out from CD44^+^/CD24^+^/ESA^+^, CD44^+^/CD24^+^/ESA^-^, CD44^+^/CD24^-^/ESA^+^, or CD44^+^/CD24^-^/ESA^- ^sorted cells. ESA, epithelial-specific antigen.

After initial seeding, all sorted phenotypes from the SUM159 line exhibit similar plating efficiency and colony formation, although unsorted, CD44^+^/CD24^-^/ESA^+^, and CD44^+^/CD24^-^/ESA^- ^populations formed more colonies than did the CD44^+^/CD24^+^/ESA^+ ^cells (*P *< 0.0003; Figure 4a, blue bars). The day 6 colonies were trypsinized and replated at the same low density for two additional rounds in order to assay for self-renewal and clonogenic potential of the cells that seeded the initial colonies. After the second round of passaging, the CD44^+^/CD24^+^/ESA^+ ^sorted cells exhibit the most depleted clonogenic potential among all the populations (*P *< 0.0001; Figure 4a, red bars). Upon the third serial replating, unsorted (*P *= 0.063) and CD44^+^/CD24^-^/ESA^+ ^sorted (*P *= 0.001) cells continue to enrich for CFUs, whereas the CD44^+^/CD24^+^/ESA^+ ^and CD44^+^/CD24^-^/ESA^- ^sorted cells exhibit evidence of exhaustion at the third passage (Figire 4a, yellow bars versus red bars). Similar results were seen upon serially replating sorted SUM149 cells (data not shown).

To assess the ability of the various populations to differentiate and reconstitute the parental cell line, SUM159 and SUM149 cells were sorted on the basis of CD44, CD24 and ESA subpopulations (Figure 4b), plated, and allowed to expand *in vitro *for 12 days. Expanded cultures were then analyzed by flow cytometry for reconstitution and differentiation (Figure 4c). The cultures seeded with CD44^+^/CD24^-^/ESA^+ ^enriched cells expand back into cultures nearly identical to the parental cell line and unsorted cultures that were seeded in parallel (Figure 4c). For the SUM149 cell line, this reconstitution and differentiation represents a change from nearly 100% CD24^- ^to 5.7% CD24^-^, whereas for the SUM159 cell line reconstitution and differentiation represents a change from nearly 100% ESA^+ ^to 1.9% ESA^+^. In contrast, cultures derived from CD44^+^/CD24^+^/ESA^+ ^cells from either cell line remained 98% CD44^+^/CD24^+^/ESA^+^.

We next examined the phenotype of the cell colonies that expanded after sorting and replating. Consistent with the fluorescence-activated cell sorting data (Figure 4c), CD44^+^/CD24^-^/ESA^+ ^enriched cells formed heterogeneous mixed colonies that best recapitulated the morphological phenotype of the parental cell line (Figure 4d). In contrast, colonies derived from CD44^+^/CD24^+^/ESA^+ ^cells exhibited a luminal type phenotype, whereas CD44^+^/CD24^-^/ESA^- ^colonies exhibited a basal type phenotype regardless of the cell line from which the cells were sorted from. These data further support the finding (Figure 1) that a basal or luminal cellular phenotype is consistent with CD44^+^/CD24^-^/ESA^- ^or CD44^+^/CD24^+^/ESA^+ ^cells. It is worth noting that CD44^+^/CD24^-^/ESA^- ^basal cells from the SUM149 line have a capacity to generate CD44^+^/CD24^+^/ESA^+ ^cells, but this differentiation is significantly less efficient than that of the stem-like cells.

The operational definition of stem cell activity is the ability to self-renew and reconstitute a phenotypically diverse population of cells. Because cancer cell lines are generally less heterogeneous than freshly isolated primary tumor cells and have the ability to grow indefinitely, in principle this property may not be maintained in established cell lines. However, these data collectively demonstrate that human breast cancer cell lines maintain a cellular hierarchy, such that a small fraction of cells enriched in the CD44^+^CD24^-^ESA^+ ^subpopulation can self-renew (as indicated by increasing serial CFUs) and can reconstitute the differentiation spectrum of the parental cell line.

### Sphere formation mimics tissue regeneration and correlates with tumorigenicity

Suspension sphere cultures have been used to enrich for and characterize stem/progenitor cells from primary breast and glioblastomic tissues [24,27,28]. This process is presumed to simulate the events of tissue regeneration and maintenance from cells that survive suspension conditions. In this process, an initial phase of symmetric expansion of the seeding stem cells precedes a phase of asymmetric division, which gives rise to the differentiated progeny that comprise the sphere bulk. To evaluate whether tumorsphere formation indeed mirrors tissue regeneration in our breast cancer cell lines, we assayed the percentages of CD44^+^/CD24^-^/ESA^+ ^cells in maturing tumorspheres. We reasoned that if each sphere is seeded and maintained by a small population of CD44^+^/CD24^-^/ESA^+ ^stem cells, then smaller spheres will contain an over-representation of CD44^+^/CD24^-^/ESA^+ ^compared with large spheres or nearly confluent adherent cultures, both of which will contain more differentiated progeny cells.

**Figure 5:**
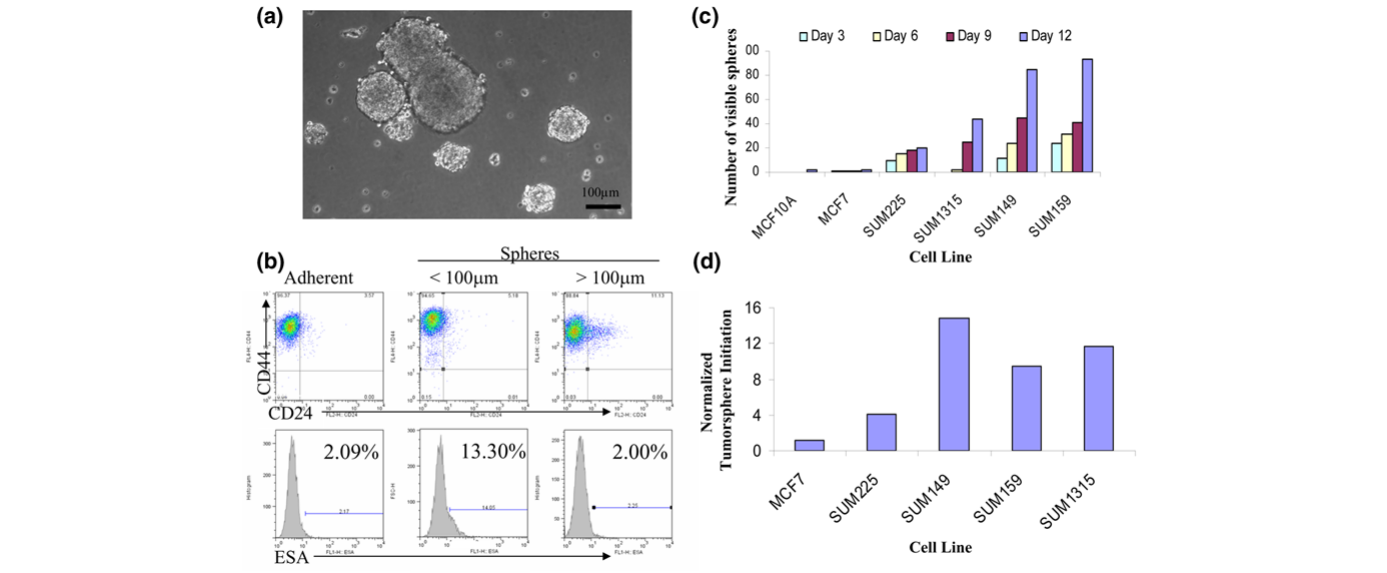
Tumorsphere formation correlates with presence of CD44^+^/CD24^-^/ESA^+ ^cells. **(a) **Phase-contrast brightfield image of SUM159 tumorspheres at day 11 under a 10× objective. **(b) **Representative flow cytometry profiles of SUM159 cells grown in adherent or nonadherent cultures. Nonadherent tumorspheres were filtered through cell strainers to separate spheres smaller than 100 μm from spheres larger than 100 μm. Percentage indicated in epithelial-specific antigen (ESA) histograms represents the population of CD44^+^/CD24^-^/ESA^+ ^cells. **(c) **For each cell line, 5,600 single cells/cm^2 ^were plated on super-low adherence plates and spheres visible by eye were counted every 3 days. **(d) **The absolute number of tumorspheres on day 12 was divided by the corresponding crystal violet value (see Materials and methods) for each cell line to correct for the proliferation rate of the cell lines.

We used cell strainers to separate spheres larger than 100 μm from smaller spheres (Figure 5a). Collected tumorspheres were dissociated into single cell suspension and analyzed by flow cytometry for the percentage of CD44^+^/CD24^-^/ESA^+ ^cells. SUM159 tumorspheres less than 100 μm contained sixfold more CD44^+^/CD24^-^/ESA^+ ^cells, whereas larger tumorspheres contained a similar percentage of CD44^+^/CD24^-^/ESA^+ ^cells to that of a nearly confluent adherent culture (Figure 5b). Comparable fold increases were also observed for the smaller SUM1315 and SUM149 tumorspheres compared with matched adherent cultures (Additional file 4 [part A]).

Next, to evaluate whether tumorsphere formation is related to the percentage of CD44^+^/CD24^-^/ESA^+ ^cells, the number of spheres formed by each cell line was quantified. The absolute number of tumorspheres that develop increases over 12 days (Figure 5c), suggesting that the proliferation rate of the cell line contributes to the number of observable spheres. Because the growth rate of suspension cultures is similar to that of matched adherent cultures (Additional file 4 [part B]), and the proliferation rates among the cell lines vary (Additional file 4 [part C]), tumorsphere formation was normalized to the proliferation rate of each cell line (Figure 5d). In doing so, it is clear that tumorsphere formation correlates with the percentage of CD44^+^/CD24^-^/ESA^+ ^cells in the cell line (Figures 5d and 2d), and thus also correlates with tumorigenicity.

### CD44^+^/CD24^-^/ESA^+ ^cells cycle more slowly and are resistant to chemotherapies

**Figure 6:**
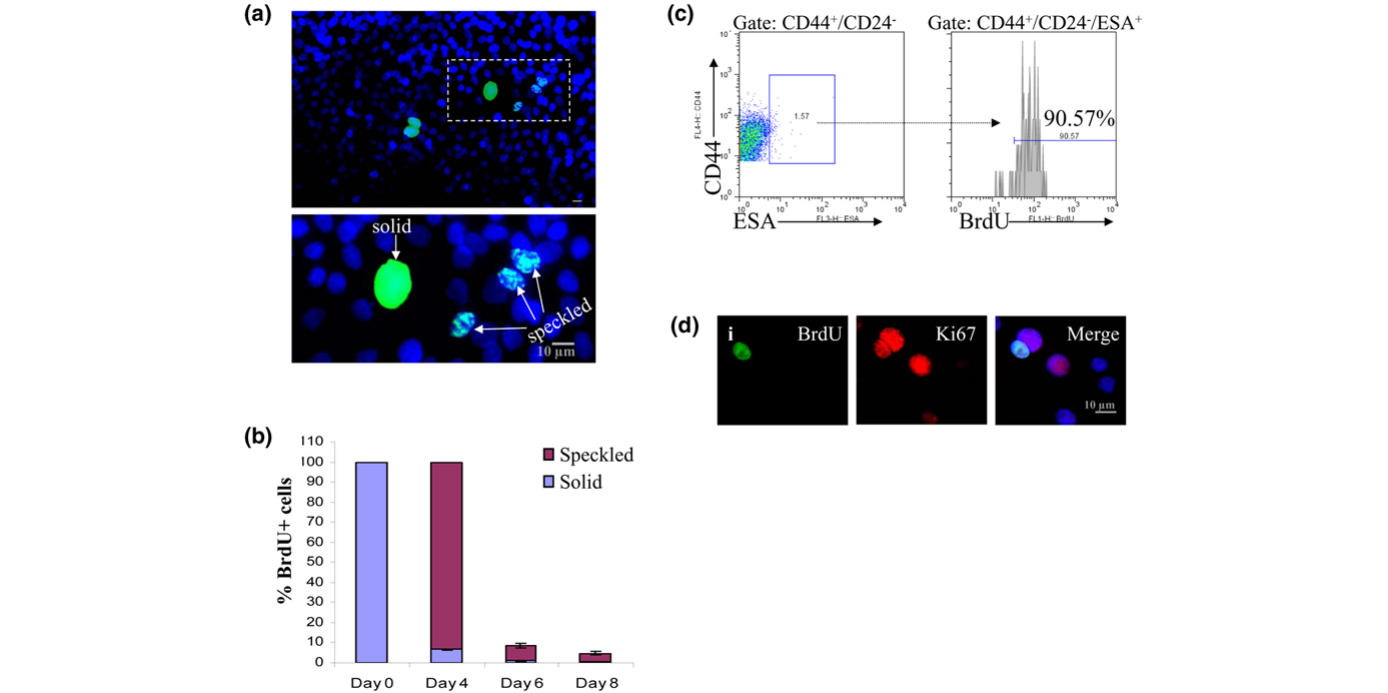
CD44^+^/CD24^-^/ESA^+ ^cells retain BrdU label. **(a) **SUM159 were continuously treated with 5-bromo-2-deoxyuridine (BrdU) for 10 days. Cells that stain for BrdU label 6 days after chase exhibit either a solid or speckled pattern of label retention: BrdU (green) or DAPI (4',6 diamidino-2-phenylindole; blue). **(b) **SUM159 and MDA.MB.231 cells were pulse-chased with BrdU. The percentage of cells with either solid or speckled BrdU staining were counted at the indicated time points. Data are presented as the mean ± standard error of the mean for at least three separate 10× fields of two separate experiments. **(c) **Proliferating cultures of SUM159 cells were pulse-chased and analyzed by flow cytometry for CD44^+^/CD24^-^/ESA^+ ^and the presence of BrdU. Note that about 90% of CD44^+^/CD24^-^/ESA^+ ^SUM159 cells retain BrdU label at 6 days after chase. **(d) **Immunoflourescence of BrdU and Ki67 in cells that were BrdU pulse-chased. (i) Co-localization of BrdU (green) and Ki67 (red); note that of the adjacent nuclei that are both Ki67 positive, only one contains BrdU. ESA, epithelial-specific antigen.

Slower cell cycle kinetics and the ability to retain DNA label are characteristics of normal tissue stem cells that have been assayed by [^3^H]deoxythymidine or BrdU incorporation [29,30]. To determine whether cancer stem-like cells in cell lines share this attribute, asynchronous SUM159 and MDA.MB.231 cells were cultured with BrdU for 10 days, allowing incorporation of BrdU label into DNA of all cells (Additional file 5 [part A]). Cells were then replated at low densities and chased with non-BrdU containing media to examine label retention at 4, 6, or 8 days after chase. After 6 days of chase, 7% of cells stained for partial BrdU label retention, seen as speckles in the nucleus, indicating that at least one round of DNA replication and division had taken place (Figure 6a,b). In contrast, only 0.9% of cells retained complete BrdU labeling, indicating a failure to divide during the chase period (Figure 6a,b). By flow cytometry, 90% of the CD44^+^/CD24^-^/ESA^+ ^cells contain BrdU label 6 days after chase, whereas again only 7% of the total population is BrdU^+ ^using the same gate (Figure 6c and data not shown). Furthermore, cells containing the highest 1% of BrdU label, corresponding to complete BrdU label retention and therefore a lack of division during the chase period, are comprised of only 30% CD44^+^/CD24^-^/ESA^+ ^cells (Additional file 5 [part B]). Cultures were immunostained for BrdU and the proliferation marker Ki67 at 6 days after chase to examine the proliferation rate of the cells. As expected, nearly all of the cells in the cultures stained positive for Ki67, indicating that most cells are in S-phase. Interestingly, 80% of the BrdU^+ ^cells also stained positive for Ki67 (Figure 6d [indicated by i]), further supporting the notion that BrdU label retention is not specific only for quiescent cells. These results indicate that CD44^+^/CD24^-^/ESA^+ ^cells do cycle but at a slower rate than the bulk of the cell line.

**Figure 7:**
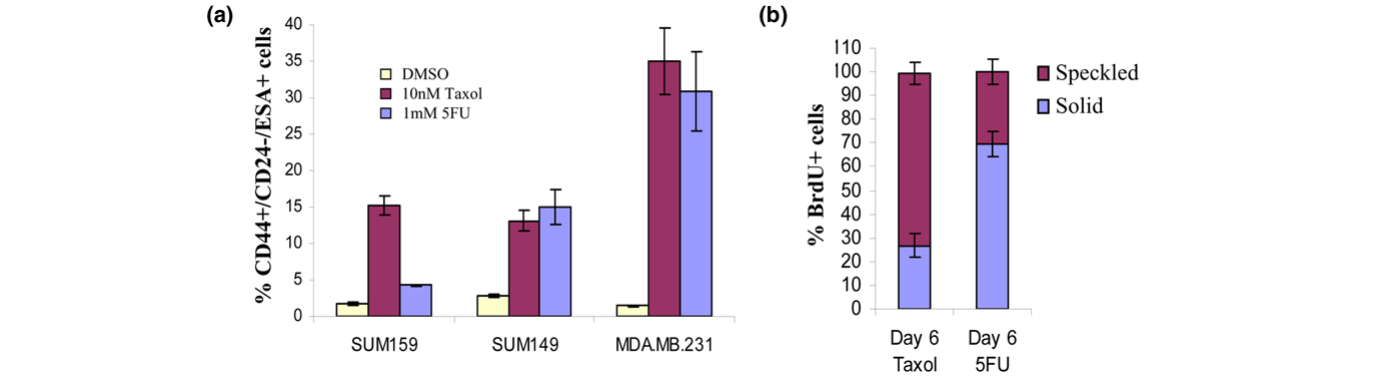
CD44^+^/CD24^-^/ESA^+ ^cells preferentially survive chemotherapy. **(a) **Quantification of viable CD44^+^/CD24^-^/ESA^+ ^cells in various human breast cancer cell lines following a 6-day treatment with 10 nmol/l paclitaxol (Taxol) or 1 mm 5-fluorouracil (5-FU). Data are presented as the mean ± standard error of the mean for four separate experiments. **(b**) SUM159 cells were continuously treated with BrdU for 10 days and chased with media containing 10 nmol/l Taxol or 1 mmol/l 5-FU. On day 6 of the chase, cells were stained for 5-bromo-2-deoxyuridine (BrdU) and counted for the solid (complete label retention) and speckled (partial label retention) staining. DMSO, dimethyl sulfoxide.

Given that breast cancer cell lines contain cells that exhibit properties of cancer stem-like cells, and cancer-initiating cells in primary human leukemia and glioblastoma are resistant to chemotherapy [31,32], we sought to determine whether cell line-derived CD44^+^/CD24^-^/ESA^+ ^cells would also preferentially survive treatment with chemotherapeutic agents. To this end, nonconfluent adherent cultures of SUM159, SUM1315, and MDA.MB.231 cells were treated for 6 days with high dose 10 nmol/l paclitaxel (Taxol) or 1 mmol/l 5-FU (Additional file 6 [part A]). Of the surviving cells, CD44^+^/CD24^-^/ESA^+ ^cells were enriched 5-fold to 30-fold in chemotherapy-treated cultures as compared with placebo-treated control cultures (Figure 7a). As expected for cell cycle inhibitory drugs, the cell line with the highest proliferation rate, MDA.MB.231, exhibited the greatest cytotoxicity (Additional files 4 [part C] and 6 [part B]), but of the 1% surviving cells after Taxol or 5-FU treatment an average of 30% or 35% were CD44^+^/CD24^-^/ESA^+^, respectively (Figure 7a).

The observation that 90% of CD44^+^/CD24^-^/ESA^+ ^cells retain BrdU label in an untreated asynchronously diving culture suggests that one mechanism by which these cells preferentially survive cell cycle inhibitory agents could be that they divide more slowly or remain quiescent compared with the bulk of the line. To examine this possibility, BrdU-labeled cultures were chased for 6 days with media containing 10 nmol/l Taxol or 1 mmol/l 5-FU. In contrast to cells chased with plain media, nearly all of the cells with intact nuclei following chemotherapy chase were BrdU^+^, yet 30% to 70% of these cells exhibited the speckled pattern of BrdU staining, consistent with the notion that they had undergone cell division. Hence, although cells that survive chemotherapy do cycle more slowly, they continue to replicate, which suggests that their resistance is not due solely to quiescence (Figure 7b and Additional file 6 [part C]). Taken together, these data show that CD44^+^/CD24^-^/ESA^+ ^cells in breast cancer lines exhibit increased resistance to chemotherapy, providing evidence for a mechanism of post-treatment recurrence.

## Discussion

Although the existence of tumor-initiating cells in solid human tumors is widely accepted, it is unclear whether cancer-derived cell lines contain similar cells. Primary carcinoma cells are presumed to be the only representatives of actual human cancers, because prolonged *in vitro *culturing is thought to result in loss of crucial properties including hierarchal organization and heterogeneity found in tumors [7]. However, we have found that established breast cancer cell lines posses a small fraction of self-renewing tumorigenic cells with the capacity to differentiate into phenotypically diverse progeny. This observation suggests that cell lines are excellent models in which to study cancer stem cells.

We found that CD44^+^/CD24^-^/ESA^+ ^cells exhibit properties of self-renewal *in vitro*, form tumors from very few cells, divide slowly, and are selectively resistant to chemotherapy, all of which are hallmarks of cancer stem cells. In light of recent studies that suggest that presence of CD44^+^/CD24^- ^cells does not correlate with clinical outcome [33] or with distant metastasis [17], we also found that cell lines with few CD44^+^/CD24^- ^cells were not necessarily less tumorigenic than cell lines with very high numbers of CD44^+^/CD24^- ^cells. However, it is important to note that in luminal cell lines that are nearly 100% ESA^+ ^(MCF7 and SUM225), sorting cells for the phenotype CD44^+^/CD24^- ^is sufficient to enrich for tumorigenic cells. In contrast, in basal cell lines with high percentages of CD44^+^/CD24^- ^cells (MDA.MB.231, SUM159, and SUM1315), sorting for ESA^+ ^cells is sufficient to enrich for breast cancer-initiating cells. Finally, in cell lines that exhibit two distinct CD44/CD24 populations (for example, SUM149), all three markers are required to enrich correctly for tumor-initiating stem-like cells.

Tumor progression is thought to result from the genetic instability within cancer cells that selects for clonal expansion and evolution of more aggressive tumor behaviors over time. However, despite continual genetic and phenotypic drift, breast cancers do not radically change their phenotype, even after many years or various therapeutic regimens. Likewise, cancer cell lines do not radically change their phenotype over many passages [9]. Although it is indeed possible to select clonogenic variants within a cell line that exhibit different genetic and biological properties from the parental population, it is widely recognized that single cell cloning is extremely inefficient, yielding few clones with the capacity to expand and propagate indefinitely. Moreover, single cell clones derived from the same cancer cell line are not equally tumorigenic or metastatic [34-36]. This observation supports the notion that not every cell within a cell line can self-renew or is equally tumorigenic, further illustrating that cell lines possess a functional hierarchy and heterogeneity akin to primary tumors.

During the course of characterizing the hierarchal dynamics of cells in culture, we observed that the percentage of CD44^+^/CD24^-^/ESA^+ ^cells within a line varied depending on various conditions. For example, cell density, the presence or absence of growth factors, and even the frequency of passaging influenced the percentage of CD44^+^/CD24^-^/ESA^+ ^cells detected in a cell line. These observations suggest that the dynamic nature of cell lines is regulated by their immediate microenvironment.

## Conclusion

Although the existence of tumor-initiating cells in solid human tumors is widely accepted, it is unclear whether cancer derived cell lines contain similar cells. Here, we show that human breast cancer cell lines exhibit a cellular hierarchy that is characteristic of primary breast tumors, in which a small population of cells with the CD44^+^CD24^-^ESA^+ ^phenotype enriches for tumor-initiating cells that self-renew *in vitro *and give rise to phenotypically diverse progeny. The fact that a small population of these breast cancer stem-like cells survives chemotherapy is consistent with the notion that the surviving tumorigenic cells are responsible for the 40% recurrence rate of invasive breast cancer. Therefore, it will be of great interest to determine whether CD44^+^/CD24^-^/ESA^+ ^cells are directly targeted by alternative therapies such as monoclonal antibody treatment or novel small molecule inhibitors. Based on our results, using breast cancer cell lines to target CD44^+^/CD24^-^/ESA^+ ^cells for drug discovery offers a highly promising, reproducible, and cost-effective means to identify therapies that prevent self-renewal or force depletion of tumorigenic breast cancer stem-like cells.

## Abbreviations

BrdU = 5-bromo-2-deoxyuridine; CFU = colony-forming unit; DMSO = dimethyl sulfoxide; ESA = epithelial-specific antigen; 5-FU = 5-fluorouracil; NOD = nonobese diabetic; PBS = phosphate-buffered saline; SCID = severe combined immunodeficient.

## Competing interests

The authors declare that they have no competing interests.

## Authors' contributions

CF designed and carried out all of the experiments, and prepared the manuscript. CK designed and prepared the manuscript.

## Supplementary Material

Additional file 1 File containing a table that provides the characteristics of cell lines *in vivo*. Click here for file

Additional file 2 File showing representative flow cytometry profiles of CD44 and CD24 expression in human breast cell lines. Click here for file

Additional file 4 (A) SUM1315 and SUM149 adherent cultures and tumorspheres smaller than 100 μm analyzed by flow cytometry for CD44^+^/CD24^-^/ESA^+ ^cells. (B) SUM159 cells were plated in parallel and grown for 2 weeks in adherent or nonadherent cultures. At the indicated times, cells were harvested and counted. (C) Crystal violet growth curves of adherent cells from various cell lines. (D) Flow cytometry histograms of SUM159 and MDA.MB.231 cells pulsed with BrdU for 10 days. Note that nearly 100% of the cells are labeled. Click here for file

Additional file 5 (A) Flow cytometry histograms of SUM159 and MDA.MB.231 cells pulsed with BrdU for 10 days. Note that nearly 100% of the cells are labeled. (B) Proliferating cultures of SUM159 cells were pulse-chased and analyzed by flow cytometry for BrdU CD44. Note that the cells containing the highest 1% BrdU label are only 30% CD44^+^/CD24^-^/ESA^+ ^cells. Click here for file

Additional file 6 (A) Representative phase-contrast brightfield images of SUM159 cultures after 6 days of exposure to indicated doses of chemotherapy. (B) Quantification of the average percent killing by 10 nmol/l Taxol or 1 mmol/l 5-FU 6 day treatments for SUM159, SUM149, and MDA.MB.231 cell lines. As shown, the percentage reflects both the number of cells that remain adherent post-treatment relative to a placebo treated control plate as determined by hemacytometer counting, and the percentage of adherent cells that do not take up PI stain as determined by flow cytometry. Error bars indicate mean ± standard error of the mean. (C) Immunofluorescence of SUM159 cells stained for BrdU (green) and DAPI (4',6 diamidino-2-phenylindole; blue) at 6 days after chase with 10 nmol/l Taxol or 1 mmol/l 5-FU. Click here for file

Additional file 3 (A) Additional representative flow cytometry analysis sorting scheme for *in vivo *injections. SUM159 cells were sorted on ESA. Dot plots show resulting CD44^+^/CD24^-/low^/ESA^+ ^or CD44^+^/CD24^-^/ESA^- ^fractions. (B) SUM149 cells were sorted on ESA. Dot plots show resulting CD44^+^/CD24^-/low^/ESA^+ ^or CD44^+^/CD24^+^/ESA^+ ^fractions. Click here for file
