# Supplementary material for: Human breast cancer cell lines contain stem-like cells that self-renew, give rise to phenotypically diverse progeny and survive chemotherapy
Source: Breast Cancer Res. 2008 Mar 26;10(2):R25. doi: 10.1186/bcr1982 (PMC2397524; doi:10.1186/bcr1982)
Supplement: Additional file 1 — File containing a table that provides the characteristics of cell lines in vivo. [file bcr1982-S1.doc]

**Supplemental Table 1: Characteristics of cell lines *In Vivo***

| **Cell Line** | **Median Tumor Onset** | **Tumor Type** |
| --- | --- | --- |
|  | *(1x10^6^ cells injected)* |  |
| HMEC | Never | N/A |
| MCF10A | Never | N/A |
| MCF7 | 8-12 weeks | Non-invasive, E2 dep. |
| SUM225 | 8-12 weeks | Non-invasive |
| SUM149 | 3-5 weeks | Stromalized, Invasive |
| SUM159 | 2-4 weeks | Invasive, Metastatic |
| SUM1315 | 3-5 weeks | Invasive, Metastatic |
| MDA.MB.231 | 2-4 weeks | Invasive, Metastatic |
