# Supplementary figures and images for: Human breast cancer cell lines contain stem-like cells that self-renew, give rise to phenotypically diverse progeny and survive chemotherapy
Source: Breast Cancer Res. 2008 Mar 26;10(2):R25. doi: 10.1186/bcr1982 (PMC2397524; doi:10.1186/bcr1982)

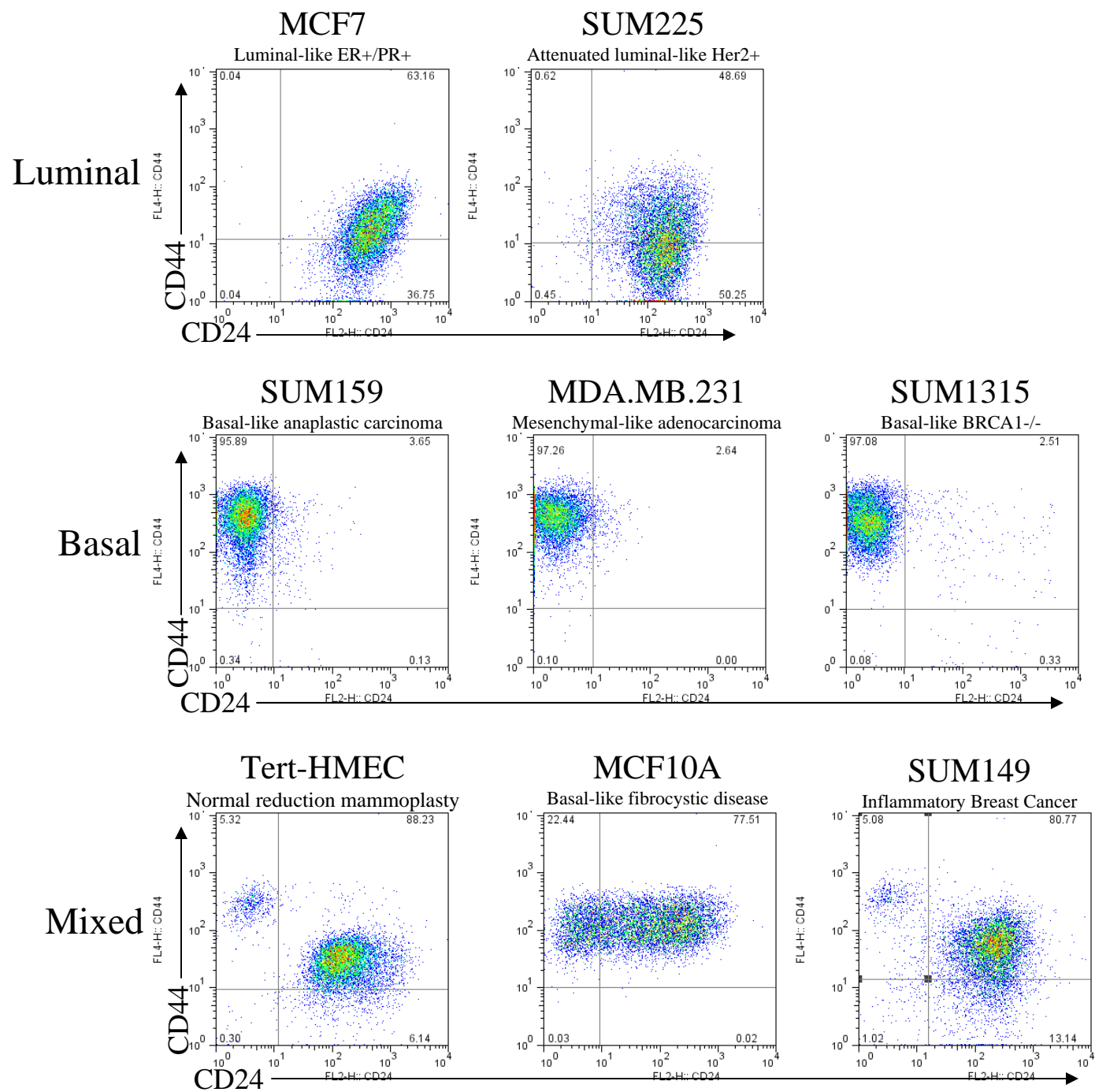

Fillmore et al. Supplemental Figure 1

Supplement: Additional file 2 — File showing representative flow cytometry profiles of CD44 and CD24 expression in human breast cell lines. [file bcr1982-S2.pdf]

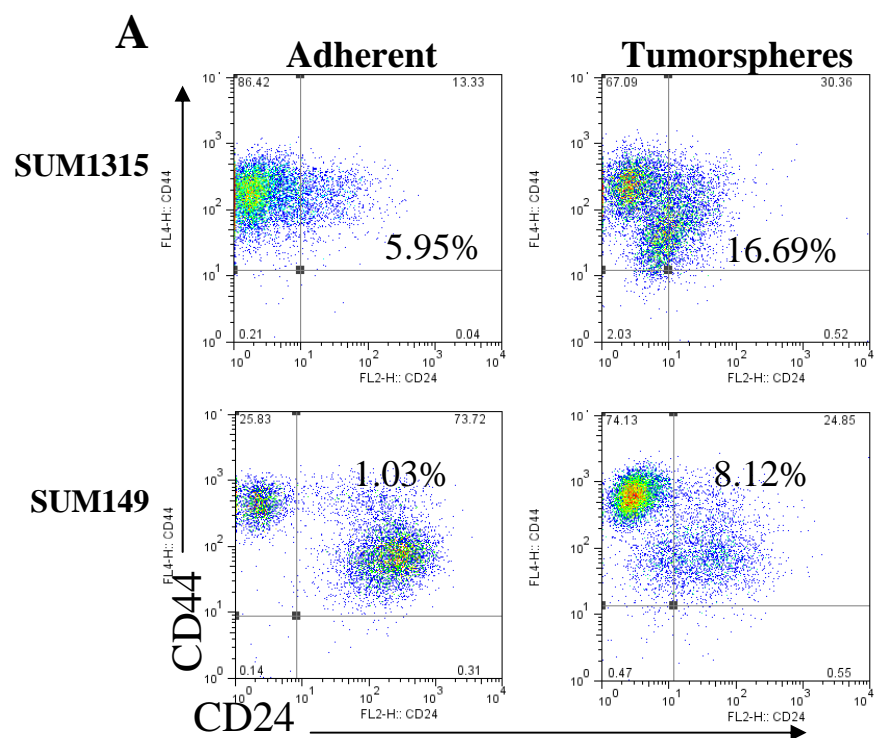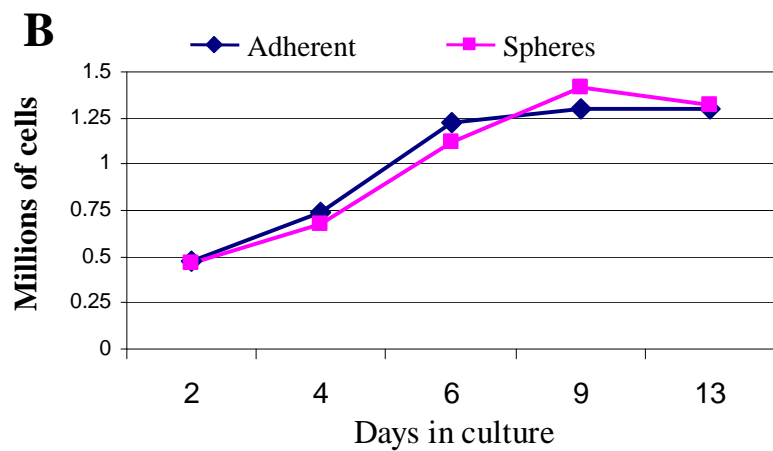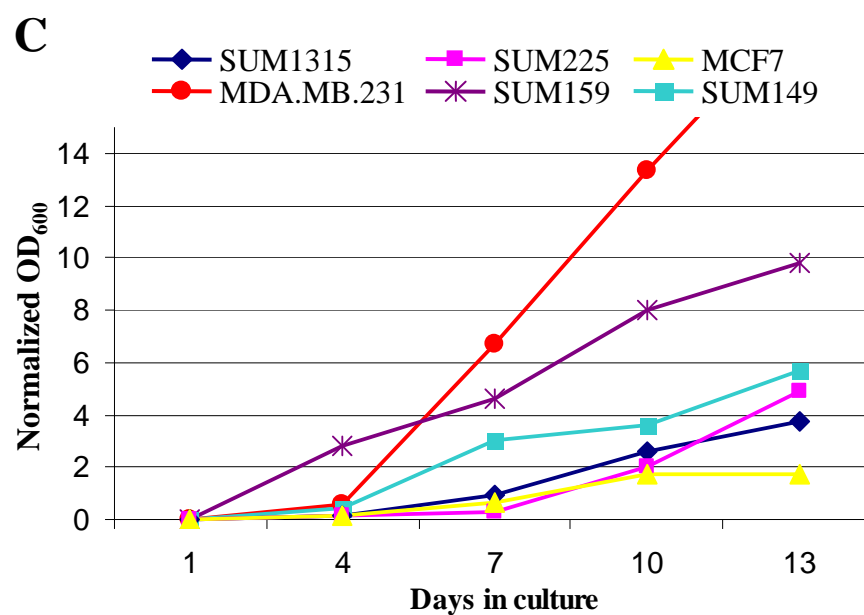

Supplement: Additional file 4 — (A) SUM1315 and SUM149 adherent cultures and tumorspheres smaller than 100 μm analyzed by flow cytometry for CD44+/CD24-/ESA+ cells. (B) SUM159 cells were plated in parallel and grown for 2 weeks in adherent or nonadherent cultures. At the indicated times, cells were harvested and counted. (C) Crystal violet growth curves of adherent cells from various cell lines. (D) Flow cytometry histograms of SUM159 and MDA.MB.231 cells pulsed with BrdU for 10 days. Note that nearly 100% of the cells are labeled. [file bcr1982-S4.pdf]

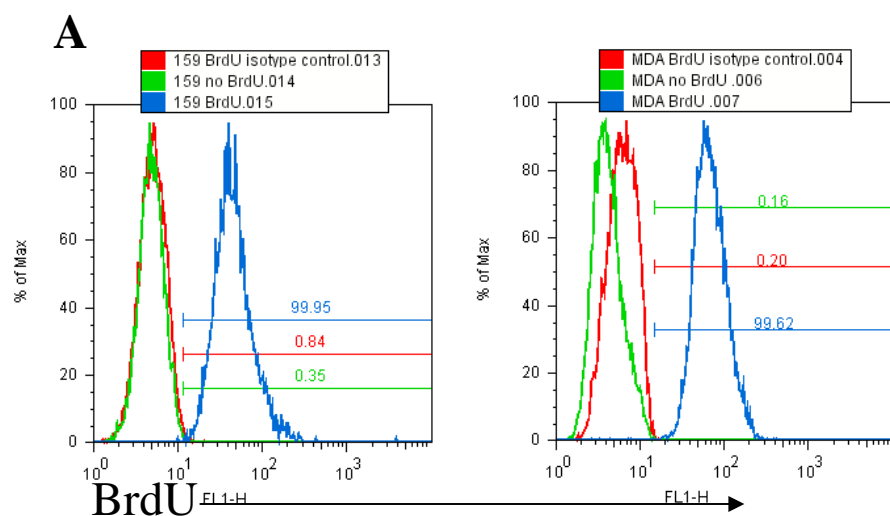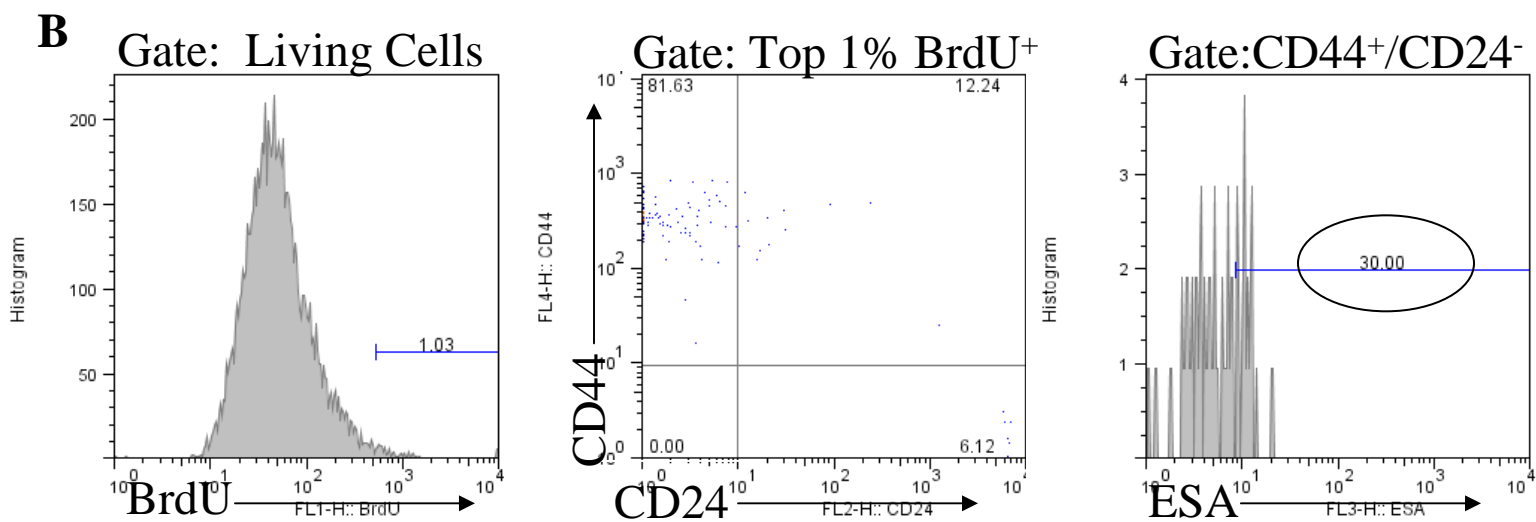

Supplement: Additional file 5 — (A) Flow cytometry histograms of SUM159 and MDA.MB.231 cells pulsed with BrdU for 10 days. Note that nearly 100% of the cells are labeled. (B) Proliferating cultures of SUM159 cells were pulse-chased and analyzed by flow cytometry for BrdU CD44. Note that the cells containing the highest 1% BrdU label are only 30% CD44+/CD24-/ESA+ cells. [file bcr1982-S5.pdf]

**A**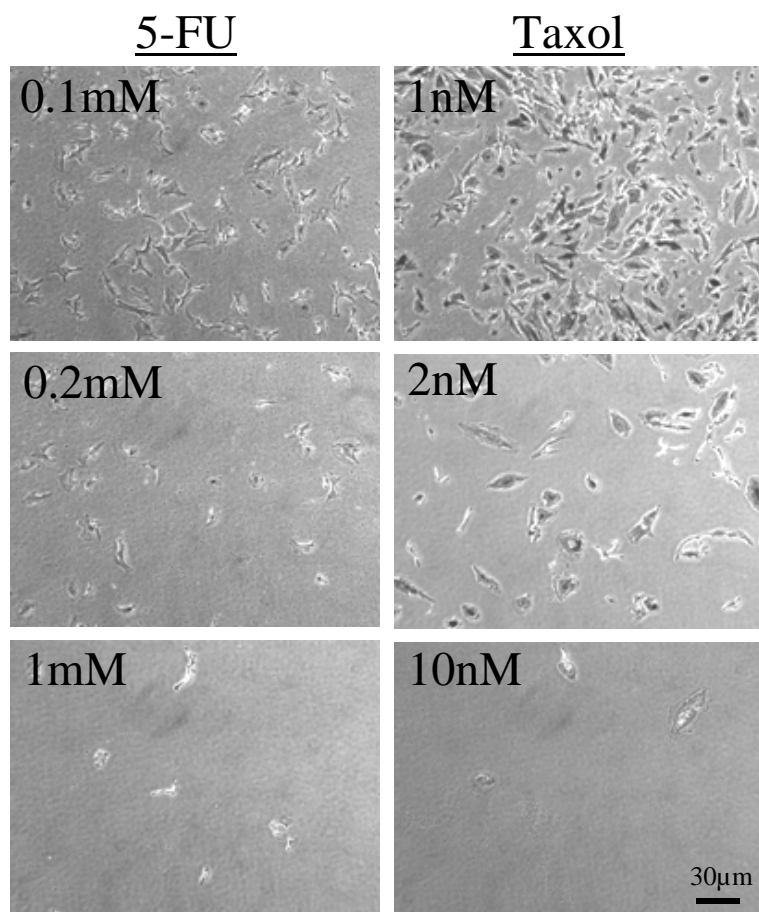**B**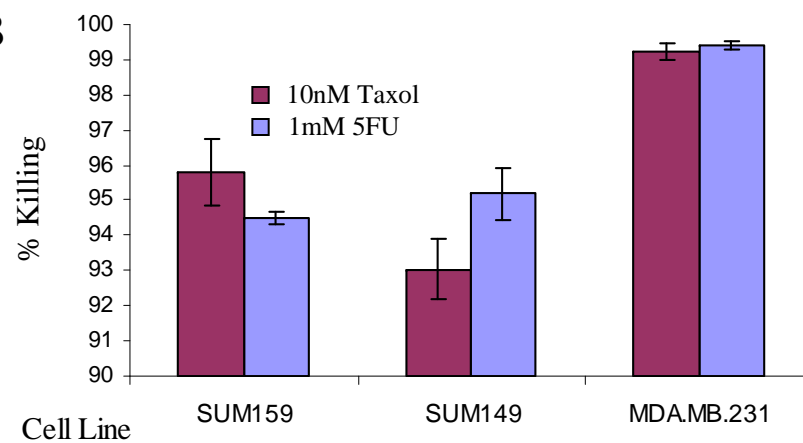**C**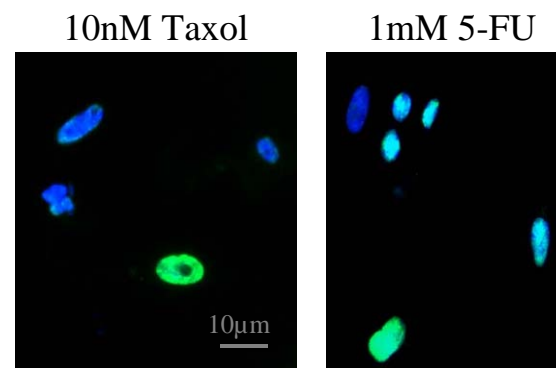

Supplement: Additional file 6 — (A) Representative phase-contrast brightfield images of SUM159 cultures after 6 days of exposure to indicated doses of chemotherapy. (B) Quantification of the average percent killing by 10 nmol/l Taxol or 1 mmol/l 5-FU 6 day treatments for SUM159, SUM149, and MDA.MB.231 cell lines. As shown, the percentage reflects both the number of cells that remain adherent post-treatment relative to a placebo treated control plate as determined by hemacytometer counting, and the percentage of adherent cells that do not take up PI stain as determined by flow cytometry. Error bars indicate mean ± standard error of the mean. (C) Immunofluorescence of SUM159 cells stained for BrdU (green) and DAPI (4',6 diamidino-2-phenylindole; blue) at 6 days after chase with 10 nmol/l Taxol or 1 mmol/l 5-FU. [file bcr1982-S6.pdf]

## SUM159

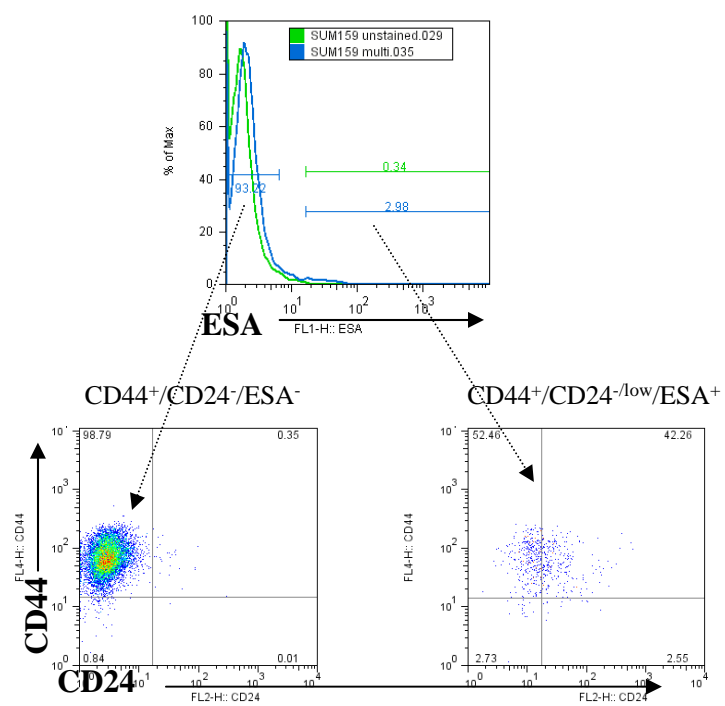

## SUM149

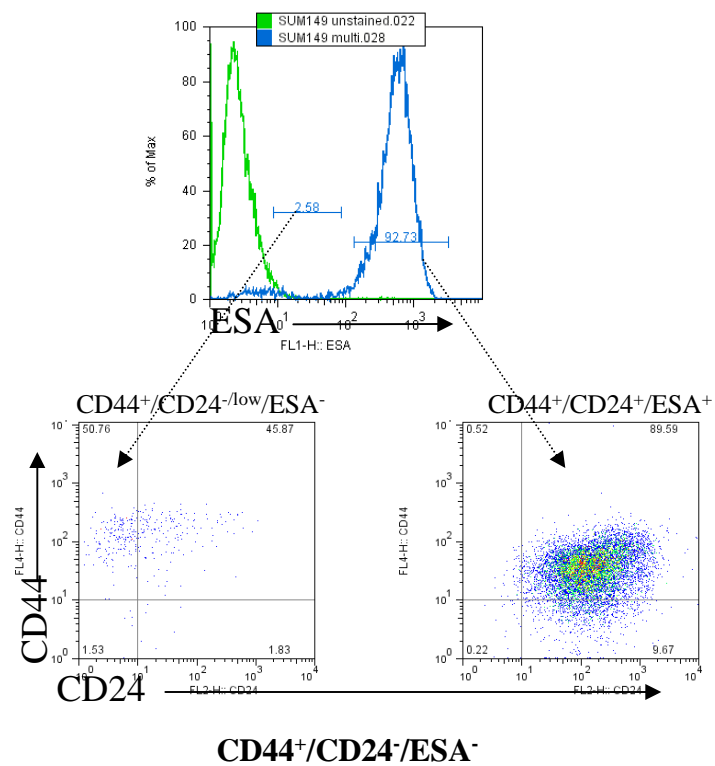

Supplement: Additional file 3 — (A) Additional representative flow cytometry analysis sorting scheme for in vivo injections. SUM159 cells were sorted on ESA. Dot plots show resulting CD44+/CD24-/low/ESA+ or CD44+/CD24-/ESA- fractions. (B) SUM149 cells were sorted on ESA. Dot plots show resulting CD44+/CD24-/low/ESA+ or CD44+/CD24+/ESA+ fractions. [file bcr1982-S3.pdf]
